# Editorial: LARC mental health summit: Suicide prevention

**DOI:** 10.3389/fpsyt.2022.1118720

**Published:** 2023-01-20

**Authors:** Kevin S. Murnane, James C. Patterson, Nicholas E. Goeders

**Affiliations:** ^1^Louisiana Addiction Research Center, Louisiana State University Health Shreveport, Shreveport, LA, United States; ^2^Department of Pharmacology, Toxicology and Neuroscience, School of Graduate Studies, Louisiana State University Health Shreveport, Shreveport, LA, United States; ^3^Department of Psychiatry and Behavioral Medicine, School of Medicine, Louisiana State University Health Shreveport, Shreveport, LA, United States

**Keywords:** addiction, suicide, Louisiana Addiction Research Center, mental health, prevention, academic medical center

This Research Topic of Frontiers in Psychiatry was developed by scientists and clinicians within the Louisiana Addiction Research Center (LARC). The LARC is one of six research centers at Louisiana State University Health Shreveport (LSUHS). LSUHS includes the School of Medicine, School of Graduate Studies, and School of Allied Health. The campus is attached to a large level-1 trauma center and safety-net hospital that serves the vulnerable communities of North Louisiana, East Texas, South Arkansas, and elsewhere. It is one of only 120 academic medical centers in the United States.

The mission of the LARC is to provide addiction research and education in an integrated environment pursuing the latest in innovative approaches and learning. Through this research it is our goal to develop therapeutic models that optimize compassionate care to patients suffering from substance use disorders (SUDs), while improving knowledge and understanding of SUDs as a public health issue through active collaboration with our community. This LARC is also focused on understanding the interaction between SUDs and critical comorbid conditions such as depression and anxiety that may contribute to drug taking. It places great emphasis on understanding how the interactions between these comorbidities and drug taking can lead to the devastating outcomes of fatal drug overdoses and suicide.

The emergence of the SARS-CoV-2/COVID-19 pandemic created a public health crisis of unprecedented magnitude. The leadership of the LARC wrote from early in the pandemic that the stress, economic insecurity, and breakdown in social support were likely to lead to or exacerbate ongoing mental and behavioral health crises, with dramatic increases in depression, anxiety, substance use, drug-related overdoses, and suicide. To combat these crises locally, the LARC organized a Mental Health Summit in Shreveport, Louisiana. Participants included local physicians and allied health clinicians, LSUHS medical residents and students, LSUHS scientists and Ph.D. graduate students, LSUHS allied health professionals and students, and LSUHS physician alumni. The goals of the summit were to educate and train scientists, providers and our community about SUDs and suicide.

In our role as a component of an academic medical center, the LARC targeted this summit to practicing and trainee scientists and clinicians, and it used an evidenced-based approach. According to the Centers for Disease Control and Prevention, during the pandemic, younger adults, adolescents, racial/ethnic minorities, and essential workers (including frontline healthcare workers) showed disproportionately worse mental health outcomes, increased substance use, and elevated suicidal ideation compared to the general population. Front-line healthcare (essential) workers were faced with shortages of protective equipment, working for long hours, fear for their lives and their families, and social isolation. Over 20% of essential workers reported seriously considering suicide during the pandemic. Adolescents and young adults were faced with disruptions of social connections during key developmental periods, disruption of their school schedule, and the breakdown in contact with friends, and over 25% reported significant suicidal ideation. Practicing psychiatrists and emergency physicians were dealing with this crisis in younger adults and essential workers with limited data with which to make evidence-based decisions, particularly as it relates to effective interventions that can be implemented while routine clinical care was hampered during the pandemic.

This Research Topic was built out of the research that was discussed during that summit. The summit was designed to understand and address the mental health consequences of the COVID-19 pandemic, particularly as it relates to stress, social isolation and substance use. The submissions discuss data on an international level, including the articles entitled “*Quality and Quantity of Serious Violent Suicide Attempts during the COVID-19 Pandemic* (Maleitzke et al.),” and “*Trends in Online Searching Toward Suicide Pre, During, and Post the First Wave of COVID-19 Outbreak in China* (Chen et al.).” The article entitled the “*Catalytic Reaction Model of Suicide* (McPherson et al.)” provides a theoretical framework for understanding suicide and the article “*Impulsiveness Indirectly Affects Suicidal Ideation Through Depression and Simultaneously Moderates the Indirect Effect: A Moderated Mediation Path Model* (Zhang et al.)” documents a domain that extensively overlaps between suicide and SUDs.

The Research Topic provides proactive tools for individuals and clinicians to use to protect adolescents, young adults, essential workers, women, underserved communities, and others from suicide and other adverse mental health outcomes. Given the social isolation necessitated by the pandemic, a major component of this was a focus on the impacts of social media use. The articles “*Social Media Use and Body Image Issues Among Adolescents in a Vulnerable Louisiana Community* (Sagrera et al.),” “*Teen Advisory Council Survey Factors Associated with Self-Harming Thoughts* (McPherson et al.),” “*Concern on Cyber Violence and Suicide during COVID-19 Pandemic* (Liu et al.),” provide detailed analyses in this regard. The Research Topic also provides strategies for protecting mental health and preventing suicide, particularly in the article “*Wesley LifeForce Suicide Prevention Gatekeeper Training in Australia: Six Month Follow-up Evaluation of Full and Half Day Community* Programs (Hawgood et al.)” and the article “*Promoting Student Wellness and Self-Care during COVID 19: The Role of Institutional Wellness* (Vazquez Morgan).” These articles also touch on strategies that can be implemented during social distancing.

The leaders of the LARC stand with individuals facing mental and behavioral health challenges. Our service and our hearts are with you. We are committed to individuals and communities that experience neglect and adverse experiences. We work every day to foster collaborative multidisciplinary translational research projects that propel new advances in science toward new treatments for mental and behavioral health. We engage our community of stakeholders throughout Louisiana and nationally/internationally to provide outreach and education on the science of addiction and the new advances in care that are emerging. We are driving innovations in the current models of care to allow for the integration of these new advances, with the goals of decreased relapse and readmission rates, decreased emergency psychiatry utilization, and increased resilience and wellbeing. It is our hope that this Research Topic can be of utility to the international scientific and medical communities as they progress toward these goals.

## Author contributions

All authors listed have made a substantial, direct, and intellectual contribution to the work and approved it for publication.

